# Influence of optical zone on myopic correction in small incision lenticule extraction: a short-term study

**DOI:** 10.1186/s12886-022-02631-4

**Published:** 2022-10-21

**Authors:** Pan Liu, Dongyu Yu, Boyu Zhang, Shiqi Zhou, Haoran Zhu, Wanyun Qin, Xinqi Ye, Xianghui Li, Yan Zhang, Ying Bai, Yuan Wang, Zhengbo Shao

**Affiliations:** 1 Department of Ophthalmology, the Second Affiliated Hospital of Harbin Medical University, No.246 Xuefu Road, Nangang District, 150086 Harbin, Heilongjiang Province China; 2 Future Medical Laboratory, the Second Affiliated Hospital of Harbin Medical University, No.246 Xuefu Road, Nangang District, 150086 Harbin, Heilongjiang Province China; 3 Harbin Medical University, No.157 Baojian Road, Nangang District, 150081 Harbin, Heilongjiang Province China

**Keywords:** Small incision lenticule extraction, Optical zones, Refractive outcomes, Myopia

## Abstract

**Background:**

To evaluate the influence of preoperative optical zone on myopic correction in small incision lenticule extraction.

**Methods:**

In this retrospective clinical study, 581 eyes from 316 patients underwent SMILE were selected, including 117 eyes in the small optical zone group (range from 6.0 to 6.4 mm) and 464 eyes in the large optical zone group (range from 6.5 to 6.8 mm). The measurements included uncorrected distance visual acuity (UDVA), corrected distance visual acuity (CDVA), spherical, and cylinder were measured preoperatively and 3 months postoperatively. Propensity score match (PSM) analysis was performed with age, gender, eye (right/left), keratometry and preoperative spherical equivalent between two different groups. The influence of optical zones on postoperative refractive outcomes were evaluated using univariate regression analysis.

**Results:**

In total, 78 pairs of eyes were selected by PSM (match ratio 1:1). There were no differences in the age, gender, eye (right/left), keratometry or preoperative spherical equivalent between the small and large optical zone groups. However, the difference of postoperative spherical equivalent was significantly between groups. Patients with larger optical zones had a trend towards less undercorrection (*P* = 0.018). Univariate linear regression model analysis found that each millimeter larger optical zone resulted in 8.13% or 0.39D less undercorrection (P < 0.001). The dependency between the optical zones and postoperative spherical equivalent was significant in the higher preoperative myopia group (*r* = 0.281, *P* < 0.001), but not significant in the lower myopia group (*r* = 0.028, *P* = 0.702).

**Conclusion:**

The diameter of optical zones would affect postoperative refractive outcomes in small incision lenticule extraction. This study indicated that larger optical zones induced less undercorrection, especially in patients with high myopia.

## Background

Small incision lenticule extraction (SMILE) is a new minimally invasive corneal refractive surgery for the correction of myopia and myopic astigmatism [1–3]. It creates a lenticule of through a 2.0 -2.5 mm incision based on the use of femtosecond laser [4]. In recent years, SMILE has been established as an effective, predictable, safe and stable refractive surgery solution [5–12]. However, overcorrection and undercorrection still exists after SMILE [1, 13, 14].

Several studies have shown that SMILE refractive outcome could be influenced by the age, gender, keratometry, preoperative spherical equivalent and optical zone [15–20]. The optical zone refers to extracted lenticule size that is designed before SMILE surgery. The scotopic pupil size, original corneal thickness and preoperative spherical equivalent are major factors to influence the clinical decision while planning the treatment zone. A refractive surgeon might prefer to design a larger optical zone to avoid the night vision complaints, such as glare, halo, and ghosting when the scotopic pupil size is relatively large [21]. However, a larger optical zone requires more corneal tissue for a given spherical equivalent refraction correction [22]. For some high myopic patients with thin cornea, a relatively smaller optical zone might be selected to save corneal tissue [23, 24]. Therefore, it’s difficult to design a proper optical zone to strike a balance between the postoperative visual quality and safety in SMILE surgery. The purpose of our study is to evaluate the relationship between optical zones and refractive outcome after SMILE for the treatment of myopia with or without astigmatism.

## Methods

### Patients

This was a comparative, retrospective clinical study. The study followed the tenets of the Declaration of Helsinki and was approved by the ethics committee of the Second Affiliated Hospital of Harbin Medical University. Patients undergoing SMILE were enrolled in this study between 2019 and 2021 in the Second Affiliated Hospital of Harbin Medical University. The inclusion criteria were as follows: 18 years or older, spherical refraction up to -9.00 diopters (D), myopic astigmatism up to 3.00 D; refraction change less than 0.50 D for the past two years, and CDVA of 20/30 or better. The exclusion criteria were the presence of active ocular disease, ocular trauma, suspected keratoconus and the expected residual stromal bed less than 250 μm. Soft contact lenses were discontinued for 2 weeks, and rigid lenses for 4 weeks before surgery.

### Surgery

All surgeries procedures were performed with the VisuMax femtosecond laser (Carl Zeiss Meditec AG) in the Second Affiliated Hospital of Harbin Medical University. The optical zone was 6.0 to 6.8 mm, and the cap diameter was 7.6 mm. The predetermined cap thickness was 100 to 120 μm, and the pulse energy ranged from 125 to 160 nJ. The side cut was placed at the 10-o’clock position of the cornea with an angle of 120 degrees and a circumferential width of 2 mm. The lenticule was extracted through the incision and all patients received 0.3% ofloxacin eye drops (Santen Pharmaceutical Co., Ltd., Osaka, Japan.) four times a day for 1 week, 0.1% fluorometholone eye drops (Santen Pharmaceutical Co., Ltd.) four times a day for 2 weeks, and 0.1% sodium hyaluronate eye drops (Santen Pharmaceutical Co., Ltd.) four times a day for 2–3 months.

### Clinical examinations

The following measurements were included preoperatively and 3 months after SMILE for all patients: slit-lamp biomicroscopy, fundus examinations, intraocular pressure, corneal topography via the anterior eye segment analysis system (Sirius, CSO, Italy), UDVA, CDVA, cycloplegic and subjective refractions. In order to evaluate the influence of optical zone on SMILE outcomes, all patients were divided into small optical zone group (range from 6.0 to 6.4 mm) and large optical zone group (range from 6.5 to 6.8 mm).

### Statistical analysis

Data analyses were performed using SPSS software version 26.0. Normality of the data was confirmed by the Kolmogorov-Smirnov test. Data are expressed as the mean ± standard deviation. The propensity score match (PSM) analysis was used to eliminate preoperative confounding factors between small and large optical zone groups. Unpaired two-tailed t test was performed to determine difference between two groups. Pearson correlation and univariate regression analyses were used to determine the relationship between optical zones and postoperative spherical equivalent. To avoid the influence of preoperative refractive status, the cohort were segregated into thirds based on preoperative spherical equivalent. Pearson correlation and univariate regression analyses were repeated in the lower third (from minimum to percentile 33) and the upper third (from percentile 67 to maximum) of preoperative SE respectively. For all cases, *P* value < 0.05 was considered to be statistically significant.

## Results

Three hundred and sixteen patients (581 eyes) were enrolled, including 165 male and 151 female. The mean age was 23.36 ± 5.48 years (range from 18 to 49 years). The clinical information of subjects is shown in Table 1.

**Table 1:** Characteristics of Patients Undergoing SMILE

Parameter	Mean ± SD
Age (y)	23.36 ± 5.48
Sphere (D)	-4.42 ± 1.52
Cylinder (D)	-0.78 ± 0.67
SE (D)	-4.81 ± 1.51
IOP (mmHg)	17.57 ± 2.55
CCT (um)	543.85 ± 26.85
Ablation depth (um)	106.36 ± 19.32
Scotopic pupil size (mm)	6.75 ± 0.74

D = diopters; SE = spherical equivalent; IOP = intraocular pressure; CCT = central corneal thickness

## Safety and efficacy

**Fig. 1:**
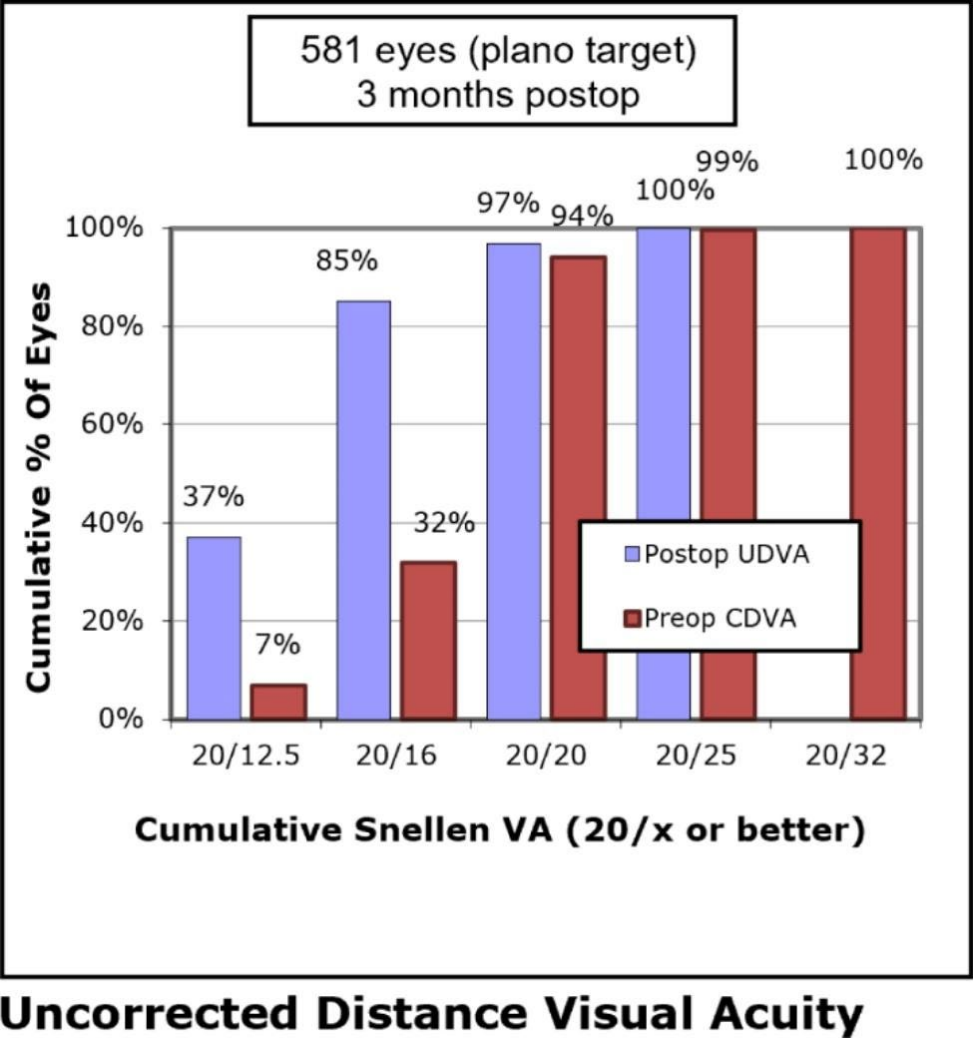
Cumulative postoperative uncorrected distance visual acuity (UDVA) and preoperative corrected distance visual acuity (CDVA) 3 months after SMILE

**Fig. 2:**
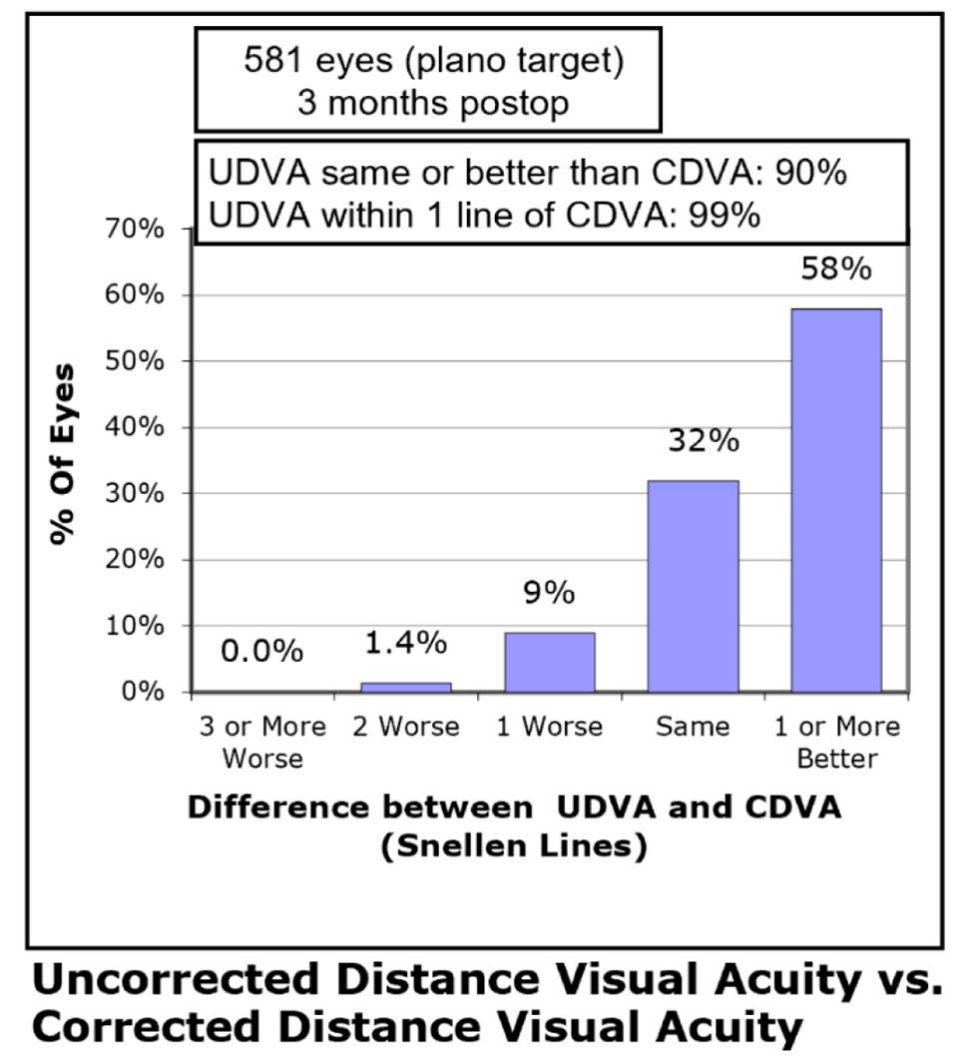
Distribution of the change in lines of postoperative uncorrected distance visual acuity (UDVA) to preoperative corrected distance visual acuity (CDVA)

**Fig. 3:**
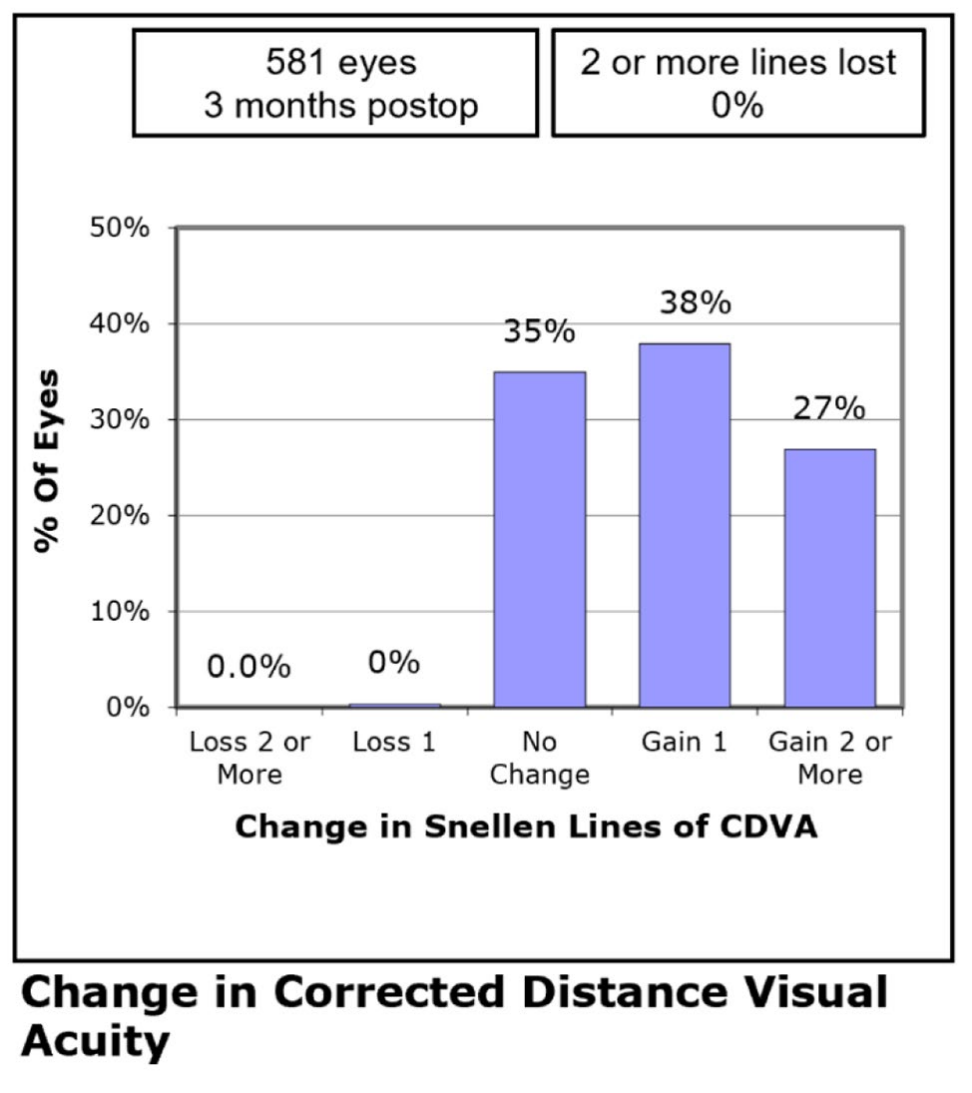
Distribution of the change in lines of corrected distance visual acuity (CDVA) 3 months postoperatively

Three months postoperatively, 563 (97%) eyes had a UDVA of 20/20 or better (Fig. 1). 521 (90%) eyes had a UDVA same or better than preoperative CDVA (Fig. 2). The mean efficacy index (ratio of postoperative UDVA to preoperative CDVA) was 1.18 ± 0.23. The CDVA remained the same in 203 (35%) eyes, whereas 376 (65%) eyes improved and 2 eyes lost one line or more at postoperative month 3 (Fig. 3). The mean safety index (ratio of postoperative CDVA to preoperative CDVA) was 1.22 ± 0.20.

## Predictability

**Fig. 4:**
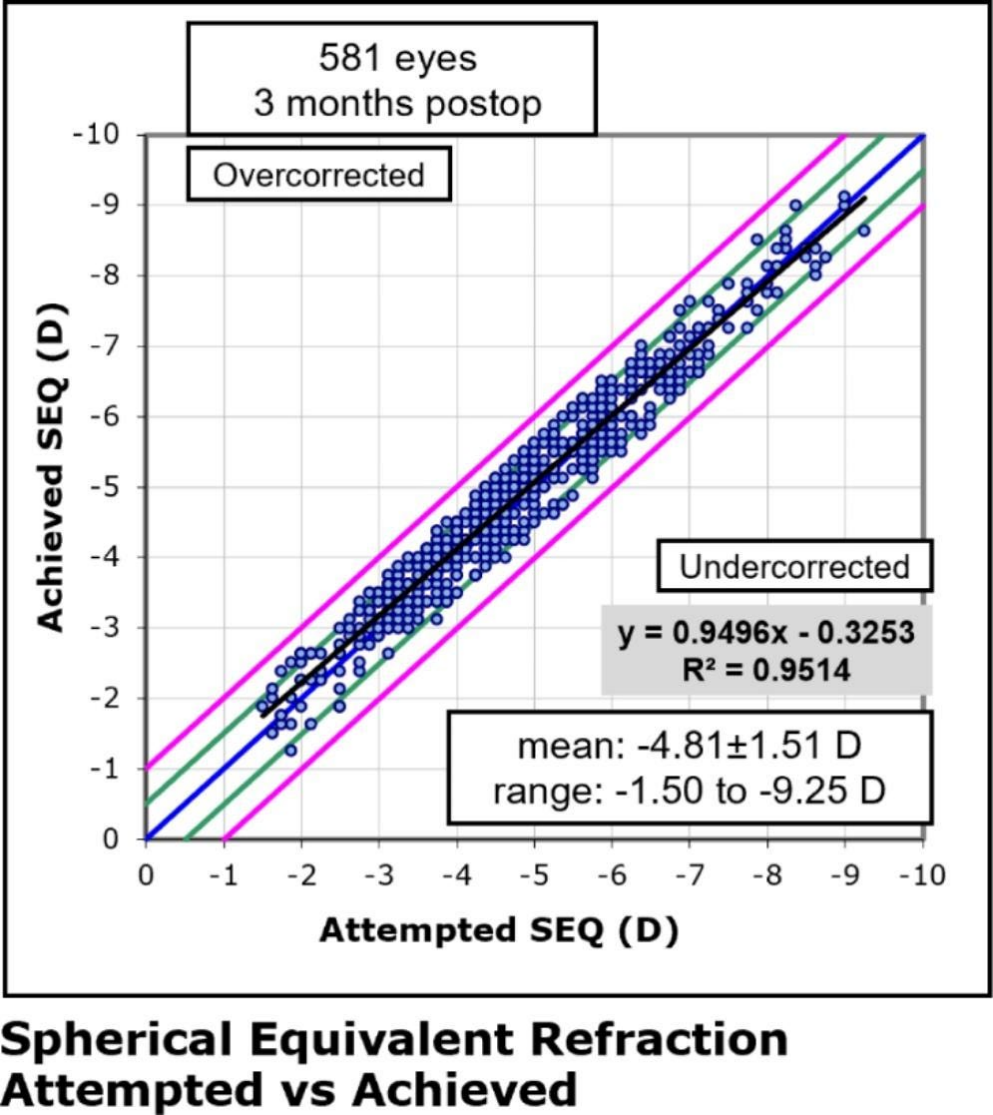
Attempted versus achieved change in spherical equivalent refraction (SEQ) 3 months after SMILE

**Fig. 5:**
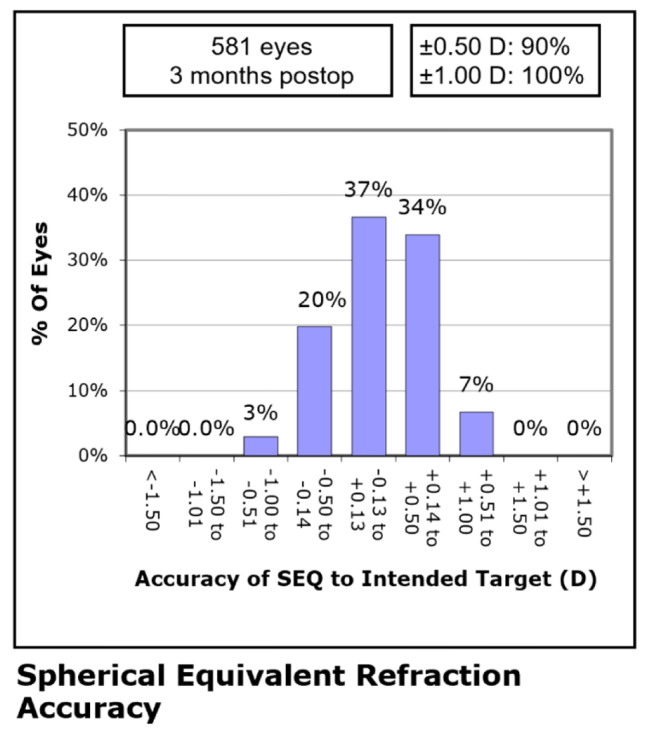
Distribution of postoperative spherical equivalent (SE) after surgery

The scatterplot of the attempted versus the achieved change in spherical equivalent refraction at 3 months after SMILE is shown in Fig. 4. The relationship between attempted and achieved correction is high with a correlation coefficient of 0.95. 525 (90%) eyes had an SE within ± 0.50 D and 581 (100%) eyes within ± 1.00D at month 3 postoperatively (Fig. 5).

## Refractive outcomes in small and large optical zone groups

There were 78 pairs of eyes matched by 1:1 PSM analysis between the small and large optical zone groups. After matching two groups for age, gender, eye (right/left), mean keratometry, and preoperative SE, the difference of postoperative SE was significantly between the small and large optical zone groups. Eyes with larger optical zone had a tendency to undercorrection at 3 months after surgery (P < 0.05) (Table 2).

**Table 2:** Clinical characteristics after matching by PSM between the small and large optical zone groups

Parameter	Small group	Large group	***P***
No. of eyes	78	78	-
Age (y)	23.54 ± 5.63	23.50 ± 5.94	0.927
Gender (M, %)	44%	45%	0.747
Eye (OD, %)	47%	47%	1.000
Preoperative SE (D)	-5.91 ± 0.92	-5.81 ± 0.99	0.484
Mean keratometry (D)	43.17 ± 1.31	43.21 ± 1.27	0.859
Optical Zone (mm)	6.23 ± 0.16	6.53 ± 0.08	< 0.001
Range	6.0 to 6.4	6.5 to 6.8	
Postoperative SE (D)	-0.10 ± 0.29	0.02 ± 0.34	0.018

SE = spherical equivalent; D = diopters

## Effect of optical zones on refractive outcomes

**Fig. 6:**
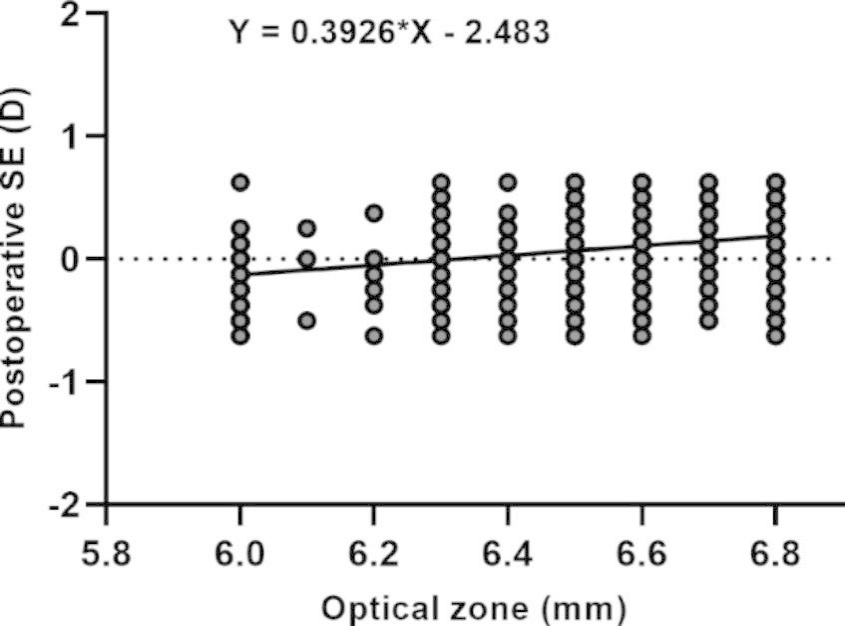
Scatterplot of the regression analysis between optical zone and postoperative spherical equivalent (SE)

The dependency between the optical zones and postoperative SE was significant (*r* = 0.247, *P* < 0.001). The patients with larger optical zones had less undercorrection. The relationship between optical zones and postoperative SE were showed in Fig. 6. Each millimeter larger optical zone resulted in 8.13% or 0.39D less undercorrection (*R*^2^ = 0.091, *P* < 0.001). The difference in postoperative SE between the lower third and upper third was significant (*P* < 0.001). The dependency between optical zones and postoperative SE was significant in the higher degree of preoperative myopia group (*R*^2^ = 0.079, *P* < 0.001), but not significant in the lower degree of myopia group (*R*^2^ = 0.001, *P* = 0.702) (Table 3). The patients with a higher degree of preoperative myopia were more influenced by optical zones.

**Table 3:** Regression analysis between optical zones and postoperative SE in different preoperative refractive status

	Preoperative SE (D)		
	**Lower Third**	**Upper Third**	
**Parameter**	**≤-4.00**	**≥ -5.38**	*P*
No. of eyes	193	194	
Preoperative SE (D)			
Mean ± SD	-3.18 ± 0.67	-6.50 ± 0.89	< 0.001
Range	-4.00 to -1.50	-9.25 to -5.38	
Postoperative SE (D)			
Mean ± SD	-0.17 ± 0.32	-0.11 ± 0.49	< 0.001
*r*	0.03	0.28	
Linear regression equation	Y= -0.07*X + 0.63	Y = 0.48*X-3.03	
*P*	0.702	< 0.001	

SE = spherical equivalent; D = diopters

## Discussion

This study designed to evaluate the refractive outcomes in eyes with small optical zone (range from 6.0 to 6.4 mm) and large optical zone (range from 6.5 to 6.8 mm) at 3 months after SMILE. The results showed that the larger optical zones induced less undercorrection, especially in patients with higher myopia.

The safety, efficacy, and predictability of SMILE in this study were comparable with most published results [5–8, 11, 18]. For safety, Kim et al. [6] reported 3.3% of eyes experienced a loss of one or more lines, 41% improved one line, and 7.2% improved two lines at 3 months postoperatively. In this study, only two eyes lost one line or more, but 38% improved one line and 27% improved two lines. Comparing efficacy, several studies [11, 15, 16] have reported that 84%, 92%, 94% of eyes had an UDVA of 20/20 or better at 3 months after surgery. In the current study, 97% of eyes had a postoperative distance UDVA of 20/20 or better. Regarding predictability, other studies [15, 16, 18] have reported a predictability of SMILE range from 70 to 100% within ± 0.50 D of target refraction, and 94–100% within ± 1.00 D. These results were similar to our findings of 90% eyes within ± 0.50 D and 100% within ± 1.00 D at the 3-month follow-up.

Previous studies [15–19] found that age, gender, eye (right/left), keratometry and preoperative spherical equivalent were the relevant factors influencing the refractive outcomes of SMILE surgery. To more accurately evaluate the influence of preoperative optical zone on SMILE surgery, the PSM analysis was used in this study. There were 78 pairs of eyes matched by 1:1 PSM analysis between the small and large optical zone groups. The differences of age, gender, eye (right/left), mean keratometry, and preoperative spherical equivalent were not significant between two groups (*P* > 0.05). However, we found that postoperative spherical equivalent was significantly different between two groups, and patients with larger optical zones had a trend towards less undercorrection (*P* < 0.05). To further verify the influence of optical zone on SMILE refractive outcomes, a univariate regression analysis between optical zone and postoperative spherical equivalent was conducted. The results showed that each millimeter larger optical zone resulted in 8.13% or 0.39D less undercorrection (*P* < 0.001).

Regarding the effect of optical zone on laser in situ keratomileusis (LASIK) refractive outcomes, Moshirfar et al. [25] conducted a retrospective cohort study of 1332 eyes underwent LASIK at 12 months postoperatively. They indicated that the 6.0 mm optical zone was more myopic postoperatively compared to the 6.5 mm optical zone in moderate myopia group. In this study, the cohort were segregated into thirds based on level of preoperative spherical equivalent. Our results shown that the effect of optical zones on SMILE refractive outcomes was significantly different on the low and high degree of preoperative myopia (*P* < 0.001). Larger optical zone resulted in less undercorrection in the upper third with the highest myopia group (*P* < 0.001). Nevertheless, the eyes with larger optical zones did not have a tendency to less undercorrection in the lower third with the lowest myopia group (*P* = 0.702). These results indicate that larger optical zone may result in less undercorrection in the eyes with high preoperative myopia, but not evident in the eyes with low preoperative myopia. Therefore, we drew a conclusion that eyes with higher preoperative myopia were more influenced by the preoperative optical zones.

The visual quality of patients after SMILE surgery would be greatly influenced by the diameter of optical zone. Although better visual quality is obtained in dark, a larger optical zone requires more corneal tissue for a given spherical equivalent refraction correction [22]. In the case of thin cornea or high myopia, a refractive surgeon would choose a relatively smaller optical zone to avoid the postoperative complication such as corneal ectasia [23, 24]. However, we found that a smaller optical zone resulted in more undercorrection, especially in eyes with high myopia in this study.

A limitation of the current study is the retrospective nature. Further, a concise follow-up period may not give a definite result while evaluating the effect of optical zone diameter on SMILE refractive outcome. It will be effective to verify the treatment method in long-term follow-ups and problematic patients. In addition, the eye sample size is relatively small in this study. Future studies with a larger sample size are essential to stablish a reliable recommendation for nomogram adjustment in patients with high myopia. Another limitation is that we only make a univariate regression analysis between optical zone and postoperative spherical equivalent. A multivariate regression analysis including more confounding factors as covariates would be beneficial to improve the accuracy of these outcomes in a future study. Moreover, the effect of optical zone on visual quality in the current study could not be assessed owing to a lack of related data. In future research, it would be of clinical importance to compare the SMILE visual quality based on preoperative optical zone diameter. In the end, the biggest shortcoming of this study is that we didn’t elucidate whether the effect of optical zone on SMILE refractive outcome was confounded by the residual stromal bed thickness. A randomized comparative study between patients with different optical zone may be effective to judge whether optical zone influence the refractive outcome independently or is confounded by the residual stromal bed thickness.

In conclusion, SMILE is a safe, effective, and predictable refractive surgery for the correction of myopia and myopic astigmatism. Our study demonstrated that the postoperative refractive outcomes of SMILE would be affected by preoperative optical zone, and the eyes with a larger optical zone have a tendency to less undercorrection. The influence of optical zones on SMILE refractive outcomes is significant in eyes with high degree of myopia, but not significant in eyes with low myopia.

## Data Availability

The datasets used or analysed during the current study are available from the first authors on reasonable request.

## Abbreviations

SMILE: Small incision lenticule extraction
UDVA: Uncorrected distance visual acuity
CDVA: Corrected distance visual acuity
LASIK: Laser in situ keratomileusis
SE: Spherical equivalent
PSM: Propensity score match
